# The association between sleep and Alzheimer's disease: a systematic review

**DOI:** 10.1590/1980-5764-DN-2023-0049

**Published:** 2024-08-26

**Authors:** Paul Nichol Galan Gonzales, Steven Gayoles Villaraza, Joseree-Ann Catindig Dela Rosa

**Affiliations:** 1 Jose R. Reyes Memorial Medical Center, Department of Neurology, Sta. Cruz, Manila, Philippines.

**Keywords:** Cognitive Dysfunction, Alzheimer Disease, Sleep Initiation and Maintenance Disorders, Sleep, Sleep Wake Disorders, Sleep Quality, Electroencephalography, Pharmaceutical Sleep Aids, Sleep stages, Disfunção Cognitiva, Doença de Alzheimer, Distúrbios do Início e da Manutenção do Sono, Sono, Transtornos do Sono-Vigília, Qualidade do Sono, Eletroencefalografia, Medicamentos Indutores do Sono, Fases do Sono

## Abstract

It is estimated that 45% of individuals with cognitive impairment experience sleep disturbances prior to the onset of cognitive symptoms. Assessing sleeping problems and enhancing sleep quality are critical first steps to reduce the risk of cognitive impairment.

**Objective:**

To review existing literature based on predefined eligibility criteria to understand the connection between sleep disturbance and Alzheimer's disease.

**Methods:**

A thorough and systematic evaluation of numerous studies was carried out to assess one or more of the following epidemiological factors: (1) sleep disorders, (2) cognitive impairment, and (3) risk estimates for cognitive impairment due to sleep.

**Results:**

Studies suggest that individuals who experience memory loss may encounter sleep disturbances before noticing other symptoms. Numerous sleep disorders, such as excessive and inadequate sleep duration, poor sleep quality, circadian rhythm abnormalities, insomnia, and obstructive sleep apnea were found to increase the risk of cognitive dysfunction and dementia. Additionally, lower sleep quality and shorter sleep duration have been linked to higher cerebral-β-amyloid levels. Objective evidence for the development of cognitive impairment is provided by the architecture of sleep stages. Patients experiencing sleep problems may benefit from specific types of sleep medicine as a preventative measure against cognitive decline.

**Conclusion:**

Sleep disorders can have adverse effects on cognitive health. The duration and quality of sleep are fundamental factors for maintaining a healthy brain as we age. Proper sleep can aid prevent cognitive impairment, particularly Alzheimer's disease and dementia.

## RESUMO

Acredita-se que 45% dos indivíduos com comprometimento cognitivo experimentem distúrbios do sono antes do início dos sintomas cognitivos. Avaliar problemas de sono e melhorar a qualidade do sono são passos críticos iniciais para reduzir o risco de comprometimento cognitivo.

**Objetivo:**

Revisar a literatura existente com base em critérios de elegibilidade predefinidos para entender a conexão entre distúrbios do sono e a Doença de Alzheimer.

**Métodos:**

Uma avaliação completa e sistemática de vários estudos foi realizada para avaliar um ou mais dos seguintes fatores epidemiológicos: (1) distúrbios do sono, (2) comprometimento cognitivo e (3) estimativas de risco de comprometimento cognitivo decorrente do sono.

**Resultados:**

Os estudos sugerem que indivíduos que experimentam perda de memória podem enfrentar distúrbios do sono antes de notarem outros sintomas. Foi constatado que vários distúrbios do sono, como duração excessiva e inadequada do sono, má qualidade do sono, anormalidades no ritmo circadiano, insônia e apneia obstrutiva do sono, podem aumentar o risco de disfunção cognitiva e demência. Além disso, menor qualidade do sono e duração mais curta do sono têm sido associadas a níveis mais altos de β-amiloide cerebral. Evidências objetivas para o desenvolvimento de comprometimento cognitivo são fornecidas pela arquitetura dos estágios do sono. Pacientes que experimentam problemas de sono podem se beneficiar de tipos específicos de medicamentos para o sono como medida preventiva contra o declínio cognitivo.

**Conclusão:**

Os distúrbios do sono podem ter efeitos adversos na saúde cognitiva. A duração e a qualidade do sono são fatores fundamentais para manter um cérebro saudável à medida que envelhecemos. Um sono adequado pode ajudar a prevenir o comprometimento cognitivo, especialmente a Doença de Alzheimer e a demência.

## INTRODUCTION

Dementia is among the significant reasons for dependence, and is a public concern that adds to the rising cost of healthcare services among the elderly^1^, with Alzheimer's disease (AD) being the most common pathology^2^. In the research by Selwood et al.^3^, findings indicate a correlation between sleep difficulties and the risk of cognitive decline and dementia. It is estimated that 45% of people with cognitive impairment encounter sleep problems before the onset of the disorder; however, current research has not yet established the exact role of sleep disorders, circadian rhythm problems, and sleep medication use in the development of dementia, especially AD.

Studies have associated several sleep disorders, including obstructive sleep apnea (OSA), insomnia, and disrupted sleep-wake cycles, to an increased risk of cognitive impairment. While an individual is asleep, the brain eliminates β-amyloid (Aβ) plaques, which are one of the primary markers of AD. Sleep deprivation can significantly raise interstitial fluid Aβ levels, further exacerbating cognitive decline^4^. Moreover, disruptions to the body's circadian rhythms have been linked to a higher risk of AD. Sleep disorders may disrupt these rhythms, leading to increased Aβ accumulation, inflammation, and oxidative stress^5^.

As the population of older adults struggling with cognitive decline and dementia continues to increase, there is a pressing need to identify risk factors and develop effective preventive measures. Numerous studies have investigated the potential correlation between sleep patterns and cognitive decline, including the risk of developing dementia. However, sleep's role as a risk factor or a confounding variable remains uncertain.

As such, this systematic review aimed to investigate the relationship between sleep disturbance and AD. Specifically, the review sought to identify potential correlations between neurological parameters such as biomarkers, sleep architecture, and sleep medications.

## METHODS

To ensure a thorough literature search, this study adhered to the Preferred Reporting Items for Systematic Reviews and Meta-Analyses (PRISMA) guidelines. Moreover, a systematic approach for conducting reviews and meta-analyses was followed using step-by-step guidance. The articles included in the analysis met at least one of the following epidemiological parameters:

- Sleep disorder;
- Cognitive impairment or dementia;
- Estimates of cognitive impairment and AD risk from sleep;
- Sleep architecture; and/or
- Sleep medication use.

Publications from PubMed, Elsevier, Research Gate, and Cochrane journals were searched through the following terms: "Sleep disorder", "Sleep medication" "Cognitive Impairment", "Electroencephalography", "Sleep stages", and "Alzheimer". In these searches, the following filters were applied: year published (2015 to present), type of study (systematic reviews, cohort studies, case reports, clinical studies), language (English), and study subjects (individuals aged 18 years and above). The reference lists and bibliographies of the papers included in this review were also manually investigated for any additional eligible research.

The inclusion criteria for this review considered publications from 2015 to 2023 in the English language, with participants who were adults 60 years of age or older; studies that were available (possible to access); and those that examined the relationship of sleep problems with cognitive impairment, biomarkers, medications, and sleep architecture. Study outcomes included wakefulness, sleep quality, sleep disorders, sleep duration, brain damage, and electroencephalogram (EEG).

However, publications prior to 2015, duplicate articles, drug trials, editorials, letters to the editor, recommendations, monographs, and dissertations were excluded, as these were articles whose subject matter did not entail a diagnosis of AD or cognitive impairment.

After eliminating duplicates, the articles from the initial search were assessed for eligibility based on their titles and abstracts by the researchers. Any studies that did not align with the review's objectives were excluded. A second screening round was conducted using the complete texts, and the final selection for the systematic review was determined afterward. References identified during the second screening round underwent a third evaluation. For thoroughness, the reference lists of included works and previous meta-analyses were cross-referenced to identify additional studies that might meet the eligibility criteria.

Data items extracted from the articles included author and year of publication, study design, study assessment or measure, study sample, and outcomes or findings.

## RESULTS

**Figure 1:**
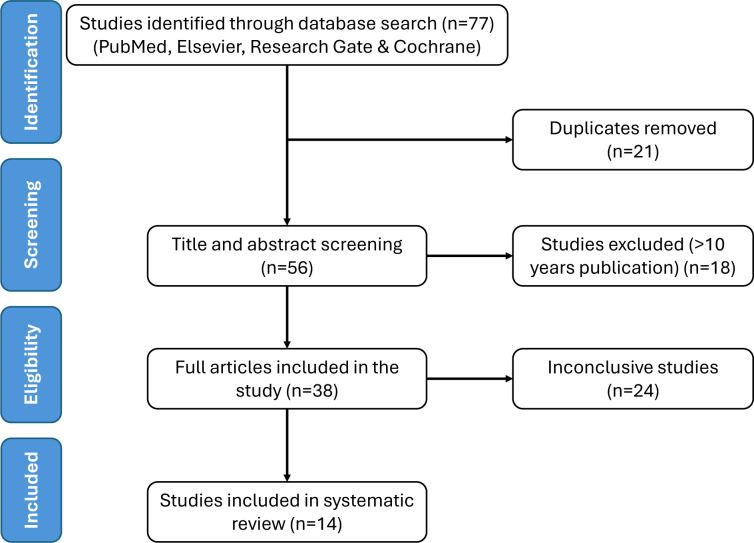
Preferred Reporting Items for Systematic Reviews and Meta-Analyses (PRISMA) flow diagram.

A total of 81 records were obtained after a literature search in PubMed, Elsevier, Research Gate, and Cochrane databases, of which 21 were removed for duplication. From the 60 records remaining, 18 were excluded due to an earlier publication date of ten years or more. Out of the 42 full-text papers that were included, 24 were deemed inconclusive due to insufficient follow-up periods, contradictory conclusions, or small sample sizes. Studies that failed to meet the inclusion criteria regarding age and diagnosis were excluded from the review. Furthermore, animal studies, incomplete or unpublished trials, and sources derived from secondary literature such as editorials, commentaries, or meta-analyses were also omitted. After eliminating duplicate entries and filtering out meta-analyses, a total of 18 articles underwent screening based on the relevance of their titles and abstracts. Subsequently, all 18 articles satisfied the eligibility criteria and were therefore incorporated into this review. The procedure employed to select the studies is delineated in Figure 1.

Table 1 ^4–21^ summarizes the results from the articles collated, including details of the authors, year of publication, study design, sleep assessment or measure, sample population, and outcomes assessed in the respective papers.

**Table 1:** Risk estimates for the effect of sleep on cognitive impairment and or dementia.

Study	Study design/quality	Sleep assessment/measure	Sample/location	Outcomes/findings
Borges et al.^16^	Systematic review	Sleep disturbance/EEG	Patients with cognitive impairment or AD	Sleep disorders may be related to AD by reduction of non-REM sleep.
Bubu et al.^5^	Systematic review	Sleep disorders/amyloid PET	Patient with and without sleep problems	Long and short sleep duration, circadian rhythm abnormality, poor sleep quality, OSA, and insomnia were associated with a higher risk of cognitive impairment and dementia than individuals who did not have sleep problems.
Burke et al.^19^	Cross sectional study	Sleep medications/observation	Patients without dementia	Sleep medications potentially mitigate the risk of cognitive impairment.
Cordone et al.^18^	Systematic review	Sleep deprivation and Aβ accumulation/EEG	Patients with sleep disturbance	Aβ accumulation in the brain is linked to the disruption of slow wave sleep.
De Gennaro et al.^17^	Case control	Sleep quality/EEG	Patients with AD	Decrease in stage 2 sleep K-complexes in AD
Fan et al.^9^	Meta-analysis	Sleep duration/questionnaire/interview	Patients with AD or dementia	Only long sleep duration is significantly associated with an increased risk of all AD and dementia.
Ferini-Strambi^13^	Systematic review	Sleep duration and sleep quality, sleep disorder/questionnaires, clinical diagnosis	Patient with and without sleep problem	Insomnia was discovered to be linked with AD, whereas sleep disorder breathing was associated with a high risk of developing dementia.
Henry et al.^8^	Mendelian randomization (MR)	Sleep duration/questionnaires, clinical diagnosis	Patient with and without sleep problems	The study's results may imply that time of sleep can develop cognitive impairment.
Insel et al.^15^	Cross sectional study	Sleep disruption/PET Regional Aβ pathology	Patients without dementia	Increased risk of Aβ deposition with reduced nighttime sleep duration occurred early, before cognitive impairment or significant Aβ deposition.
Kinugawa et al.^12^	Cross sectional/medium	Brain damage/MRI	Normative aging participant	OSA was significantly connected to the development of dementia and other related disorder.
Lim et al.^10^	Prospective cohort/high	Sleep fragmentation/wrist actigraphy	Community dwelling elderly	Sleep fragmentation is associated with dementia and cognitive impairment in older adults.
Lucey^14^	Review	Sleep questionnaires, logs, actigraphy, polysomnography, EEG	Older adults, varying cognitive statuses, with/without AD pathology	Sleep disturbances could be markers for AD pathology or a mechanism mediating increased AD risk.
Mander et al.^20^	Systematic review	Sleep disruption/EEG	Cognitively healthy adults	Sleep disruption is an early diagnostic biomarker of AD risk.
Musiek et al.^21^	Systematic review	Sleep disturbances/questionnaires, clinical diagnosis	Patients with AD	Therapeutic strategies for sleep may delay AD.
Shi et al.^7^	Systematic review	Sleep disorder/self-reported symptoms, questionnaires, clinical diagnosis, or objective monitored sleep parameters	Patient with and without sleep problem	Sleep disorder and sleep disturbance influence the development of dementia, and it also highlights the importance of quality sleep.
Spira et al.^4^	Cross sectional/medium	Sleep quality/Aβ PET	Normative aging participant	Lower sleep quality and shorter sleep are also connected with more significant Aβ burden, and it also increases the risk of dementia and AD.
Sterniczuk et al.^6^	Secondary analyses of sleep-related measure	Sleep disturbance/questionnaire	Men and women with and without experience of sleep disorder	A person who lacks sleep regularly can also increase the risk of dementia.
Zhou et al.^11^	Cross sectional/medium	Sleep disorder/questionnaire	AD patient	The severity of sleep disorders is connected with the significance of cognitive decline in AD.

Abbreviations: EEG, electroencephalogram; AD, Alzheimer's disease; REM, rapid eye movement; PET, positron emission tomography; OSA, obstructive sleep apnea; Aβ, beta-amyloid; MRI, magnetic resonance imaging.

## DISCUSSION

This review presents various prevalence ranges of sleep disturbances, cognitive impairment, and AD. Several aspects of sleep, such as disturbances, quality, duration, medication, and disorders, were primarily evaluated using questionnaires. Other assessments were carried out using wrist actigraphy, positron emission tomography (PET) scans, EEG, and magnetic resonance imaging (MRI).

### Presence of sleep disturbance and the risk of developing cognitive impairment and Alzheimer's disease

A study by Sterniczuk et al.^6^ determined the risk of dementia in relation to disturbed sleep. The study evaluated self-reported sleep problems of 17,656 individuals aged 50 and older who did not have dementia or AD at baseline. Follow-up over a 4-year period using individual sleep-related questionnaires was done. Sleep problems were identified as a significant risk factor for dementia and mortality, according to the research findings. The association was determined to be strong showing an odds ratio (OR) of 1.23 (95% confidence interval (CI) of 1.11–1.36), and persistence throughout the dementia course, indicating that sleep problems may have existed prior to the onset of other typical symptoms, such as memory loss. Moreover, Shi et al.^7^ reviewed the predictive roles of overall sleep problems in incident dementia of all causes. According to the study, individuals who suffer from sleep disturbances have a 1.19-fold higher risk of developing all-cause dementia compared to those who do not. Data showed a relative risk (RR) of 1.19 (95%CI 1.11–1.29, I2=76.2%), and p<0.001. The study also revealed that insomnia was a predictor for AD, but not for all-cause dementia.

A population-based cohort study conducted an instrumental variable analysis to examine the potential causal relationship between sleep duration and cognitive outcomes^8^. The research discovered that inadequate and excessive sleep durations were linked with reduced visual memory (p for non-linearity 3.44e-9) and slower reaction time (p for non-linearity 6.66e-16). The study suggested that the underlying relationship may not be linear and that inadequate and excessive sleep periods may both have adverse effects on cognitive abilities. As such, it was revealed that sleep duration could be a potential causal mechanism for cognitive decline. Furthermore, a study conducted on individuals who slept for an extended period (8–9 hours) had a 77% higher risk of developing all-cause dementia (hazard ratio [HR] 1.77, 95%CI 1.32–2.37) and a 63% higher risk of AD (HR 1.63, 95%CI 1.24–2.13). Conversely, no significant association was found between inadequate sleep duration (defined as <5–6 hours) and an increased risk of all-cause dementia (HR 1.20, 95%CI 0.91–1.59) or AD (HR 1.18, 95%CI 0.91–1.54). Thus, an extended duration of sleep is associated with dementia and AD, but this link may not be causative^9^.

In another study, 737 community-dwelling older adults were cross-sectionally monitored for six years to investigate the correlation between sleep fragmentation, defined as repetitive short interruptions of sleep using actigraphy recordings, and the risk of AD and cognitive decline. The data demonstrate a positive correlation between sleep fragmentation and an elevated risk of AD (HR 1.22, 95%CI 1.03–1.44, p=0.020 per one standard deviation increase in sleep fragmentation). Moreover, individuals with high levels of sleep fragmentation are 1.5 times more likely to develop AD than those with low levels. Furthermore, poor self-reported sleep quality, reduced sleep efficiency, longer wake episodes, and increased interruptions are all associated with later cognitive deterioration^10^.

A study of 176 participants with AD and controls found that sleep disorders were prevalent in 55.9% of newly diagnosed AD patients (n=84) who did not receive medication and in only 15.2% of the healthy control group (n=92). There is an indicated negative correlation between the Pittsburgh Sleep Quality Index (PSQI) score and Mini-Mental State Examination (MMSE) score (r=-0.600, p<0.010) in AD patients. This finding implies a connection between the intensity of sleep disturbances and the magnitude of cognitive deterioration in AD^11^.

### Sleep disturbance and biomarkers

Bubu et al. conducted a study that examined the relationship between sleep and preclinical AD through the use of objective measures of AD pathology, including PET scans for Aβ load^5^. The findings revealed that sleep disturbances were not only associated with cognitive impairment or symptomatic AD but also with changes in AD biomarkers that could predict those predisposed to develop AD in the future. Sleep disorders, such as OSA, were a significant risk factor for AD. One possible explanation was that hypoxia was crucial in this process and enhanced the production of Aβ, which promoted the AD development. Furthermore, OSA with cognitive impairment shared some characteristics with AD such as the genetic predisposition to APOE ε4, decrease in hippocampal gray matter, and synaptic plasticity disorders^12^. In addition, insomnia was linked to a process of systemic inflammation by activating microglial cells, which leads to the accumulation of Aβ^13^. This lack of sleep may also have an increase in phosphorylated tau forms, which are observed in the initial phases of AD development^14^.

Circadian rhythm disruption has been associated with Aβ burden. Spira et al. (2013) conducted cross-sectional research employing data from the neuroimaging sub-study of the Baltimore Longitudinal Study of Aging^4^. The sample consisted of 70 adults and aimed to measure Aβ burden. The researchers detected Aβ burden using in vivo carbon 11-labeled Pittsburgh compound B (PiB) and PET scans. According to findings, individuals who reported sleeping for shorter durations were more likely to have a greater Aβ burden. The study categorized participants into three groups based on their self-reported sleep duration; those who slept more than 7 hours showed the least Aβ burden, while those who slept less than 6 hours had the most, and the participants who slept between 6–7 hours demonstrated an intermediate level of burden. The study hypothesized that wake-related increases in neuronal activity promote the generation of Aβ peptide, which can worsen over time among individuals who experience sleep deprivation.

According to Fan et al. (2019), there is a correlation between extended sleep—defined as eight hours or more—and a higher risk of all-cause dementia (HR 1.77, 95%CI 1.32–2.37], AD (HR 1.63, 95%CI 1.24–2.13], and inflammation^9^. The study further suggests that individuals who sleep for longer durations may have underlying health concerns, such as depression, that increase their likelihood of developing dementia. Further, there is a correlation between elevated levels of interleukin (IL)-6 and C-reactive protein (CRP) and chronic low-grade inflammation, which, in turn, increases the risk of all-cause dementia by 37% and 40%, respectively. Moreover, it can also lead to a higher risk of vascular dementia and AD.

In a study conducted by Insel et al., 4,425 participants aged 65 years or older, without any cognitive impairment, were evaluated to objectively measure their sleep-wake cycles and the association between sleep and Aβ pathology^15^. The work revealed that an additional hour of sleep during the night led to a considerable reduction in both global and regional Aβ positron emission tomographic standardized uptake value ratio (95%CI, p=0.030, p<0.001). In contrast, the study found no correlation between daytime sleep and global Aβ (95%CI, p=0.700) but found association with an increase in regional Aβ (95%CI, p=0.030, p=0.001). These results suggest that adequate sleep during the night could offer significant protection against early Aβ accumulation, a fundamental characteristic of AD. Additionally, the study identified a link between daytime sleep and increased Aβ deposition in specific regions of the brain, including the precuneus and posterior cingulate, known to be among the initial sites where Aβ accumulates in the brain.

### Sleep stages and electroencephalography

Wakefulness was frequently heightened in AD, and nocturnal sleep was more disrupted due to a less synchronized circadian rhythm^16^. Alterations in the brain regions, such as the prefrontal region in non-REM (rapid eye movement) frequencies in the elderly were investigated. In percentage, stages 1 and 2 of sleep were higher when compared to slow-wave stage (SWS) 3 sleep. As individuals age, sleep spindles occur less frequently in the frontal lobe regions, which can negatively impact procedural learning. In AD patients, there is a significant decrease in K-complex (KC) density during stage 2 non-REM sleep. KC is involved in the memory consolidation process, and its disruption could be a marker for cognitive decline, as measured by MMSE scores (β=0.54; partial r=0.50)^17^. This finding supported the notion that disrupted sleep-related memory processes, specifically the reduced ability to produce KCs, may have contributed to cognitive deterioration in AD patients. Furthermore, the extent of Aβ accumulation in the brain, particularly in the prefrontal cortex and hippocampus, has been linked to the disruption of non-REM SWS contributing to impaired consolidation and cognitive decline as seen in individuals with AD^18^.

Studies indicate that during REM sleep, there is a marked rise in delta and theta waves, while faster alpha and beta waves experience a significant reduction. This phenomenon has been associated with memory consolidation and information processing. However, abnormalities on the EEG during REM sleep could potentially play a role in the onset and progression of AD.

### Sleep medications and cognitive impairment

A study investigated possible links between the use of general-category sleep medications (such as doxepin, estazolam, temazepam, trazodone, triazolam, zaleplon, and zolpidem) and mild cognitive impairment (MCI) within a cohort of 8,043 older adults, aged 71.62 years average, from Alzheimer's Disease Centers (ADC). The research aimed to determine if sleep medication mediates the risk of progression to MCI among individuals with sleep disturbance and/or APOE ε4 carriers. The study spanned over an 11-year follow-up period and found that among participants who used sleep medications, the HR for sleep disturbance was 1.14 (95%CI 0.69–1.90), suggesting no significant association with MCI^19^. However, according to this study, individuals who did not use sleep medication had a higher risk for sleep disturbance (HR 1.42, 95%CI 1.14–1.78). This suggests that sleep medication may have a protective effect against MCI in those with sleep disturbance. GABA-targeting medications currently available may indeed improve sleep quality. However, the EEG patterns they induce might not be conducive to optimal memory consolidation; in fact, these patterns might even hinder it^20^.

Other treatment options for sleep were reviewed by Musiek et al.^21^. It was revealed that melatonin supplementation may slightly enhance cognitive performance in patients with MCI. However, the efficacy of this supplement in restoring diurnal rhythm in AD patients was mixed. Meanwhile, it was revealed that bright-light therapy can enhance the stability of diurnal rhythms in those with dementia, though this treatment may not be as effective for AD patients when used alone. Orexin receptor antagonists, such as almorexant and suvorexant, have shown the potential to improve sleep and circadian disturbances in AD patients. These interventions could have implications for delaying or preventing the development of the disease.

In conclusion, the present study aimed to examine the correlation between sleep problems and the likelihood of developing dementia, particularly AD, and identified that sleep issues may occur before other typical symptoms manifest. Both insufficient and excessive sleep duration were associated with poorer cognitive outcomes. The impact of factors like depression, anxiety, and medication usage on this relationship was also observed. Sleep disorders such as insomnia, circadian rhythm disruption, and OSA might contribute to cognitive decline by promoting Aβ accumulation. EEG recordings revealed the effects on various sleep stages, providing objective evidence for cognitive decline. Lastly, sleep medication intake among those with sleeping problems may help prevent cognitive impairment by improving sleep quality. This study emphasizes the significance of sleep quality in preventing or developing AD and other cognitive impairments.

The study may have limitations related to heterogeneity in study designs, which could pose difficulties synthesizing findings because of variations in methodologies and quality. Additionally, variability in sleep assessment methods, such as self-reported questionnaires, actigraphy, and polysomnography, may introduce heterogeneity and complicate comparisons between studies. Further, it may be challenging to prove causation because there are not enough longitudinal researches looking at the temporal association between sleep disruptions and AD onset or progression. Investigating the underlying mechanisms and pathways is crucial for establishing a relationship between sleep disturbances, cognitive impairment, and AD. This research could offer valuable insights into potential methods to prevent cognitive decline and enhance sleep quality among individuals at risk of developing AD. A credible approach would be to examine the impact of sleep disruption on specific biomarkers in the brain that are involved in the pathogenesis of cognitive decline and AD. Furthermore, studies could explore specific interventions, both pharmacological and non-pharmacological, to improve sleep quality or duration to prevent or delay the onset of cognitive impairment and AD.
